# Is It Possible to Prevent the Thanatogenetic Processes in Premature Babies?

**DOI:** 10.3390/clinpract14050144

**Published:** 2024-09-02

**Authors:** Sinziana Andra Ghitoi, Mariana Deacu, Mariana Aschie, Manuela Enciu, Anca Florentina Mitroi, Georgeta Camelia Cozaru, Antonela Anca Nicolau, Cristian Ionut Orasanu, Oana Andreea Ursica, Raluca Ioana Voda

**Affiliations:** 1 Clinical Service of Pathology, Department of Pathology, “Sf. Apostol Andrei” Emergency County Hospital, 900591 Constanta, Romania; 2 Faculty of Medicine, “Ovidius” University, 900470 Constanta, Romania; 3 Department of Anatomy, Academy of Medical Sciences of Romania, 030171 Bucharest, Romania; 4 Center for Research and Development of the Morphological and Genetic Studies of Malignant Pathology (CEDMOG), “Ovidius” University, 900591 Constanta, Romania; 5 Clinical Service of Pathology, Department of Genetics, “Sf. Apostol Andrei” Emergency County Hospital, 900591 Constanta, Romania

**Keywords:** APGAR, preterm, prevention, stillborn

## Abstract

Preterm births comprise all pregnancies coming to an end before the gestational age of 37 weeks and remain the leading cause of death in children under 5 years old despite efforts to reduce their occurrence. We aim to analyze all morbidity and mortality data to understand causes and risk factors, helping in prevention efforts. This study includes 140 cases collected during 2018–2022. Demographic, maternal, and thanatogenetic data were statistically analyzed. We observed an upward slope of stillborn babies. In the case of live-born premature, the average survival was 301.76 h. The multivariate analysis noted that extremely low birth weight (HR = 5.141) and very low birth weight (HR = 4.177) are risk factors involved in mortality. Increased parity was associated with premature births with low and very low birth weight (*p* = 0.019). We observed that a mother’s age of over 30 years is predictable for the development of pregnancy-induced hypertension. Cerebral and pulmonary hemorrhages were the most common intermediate morbid conditions, with prematurity and plurivisceral hemorrhages serving as their root causes. We have identified that anthropometric measurements have a high predictability on malformed babies. The identified associations indicate a shared mechanism for certain lesion processes, which can help optimize resources for predicting and preventing preterm neonatal issues.

## 1. Introduction

Preterm births, defined as pregnancies ending before 37 weeks of gestation, can pose serious risks to both the mother and the baby. In 2020, approximately one out of 10 births were premature, resulting in over 13 million premature babies, with approximately 900,000 babies dying from complications due to prematurity [1]. In 2019, at a global level, about 2 million babies were stillborn (at a minimum of 28 gestational weeks) at widely variable rates across the world [2]. These “too soon” babies have some anthropometric features, the most important of which is birth weight. This parameter is currently used by the World Health Organization (WHO) as a definition criterion: low birth weight (LBW) is a birth weight of less than 2500 g, being nowadays classified as very low birth weight (VLBW < 1500 g) and extremely low birth weight (ELBW < 1000 g) [3].

Prematurity continues to be the leading cause of death among children under the age of 5. In recent years, there has been a significant decrease in the number of deaths, and this positive development is directly attributed to the concerted efforts of various organizations and governments across different platforms during the period of the Millennium Development Goals (MDGs) from 2000 to 2015 [4,5]. The global mortality rate in this specific population still significantly exceeds the target of 25 deaths per 1000 live births as previously defined by the Sustainable Development Goal (SDG 3.2) [6].

Available data show that approximately 90% of preterm births occur in low- and middle-income countries, primarily in Africa and South Asia. However, it is worth noting that with the advancements in assisted reproductive techniques, even well-developed countries are experiencing a rise in the occurrence of multi-fetal pregnancies. This type of pregnancy comes with an increased risk of prematurity and associated mortality [7,8,9]. Understanding and surveying the global burden of preterm birth and disparities in prevalence and mortality associated with this condition has critical importance for the appropriate usage of resources to achieve the United Nations Sustainable Development Goal target #3.2 (which aims to end all preventable deaths of newborns and children younger than 5 years old by 2030) [10,11,12,13].

The main risk factors reported for premature birth are represented by maternal age <20 years, preeclampsia, premature rupture of membrane, chronic medical illness during pregnancy, a history of stillbirth, and previous cesarean births [14,15]. Thus, according to Fuchs F et al., maternal ages under 24 and over 35 present a significantly higher risk for premature birth [16]. Also, among the main maternal pathologies that influence the evolution of pregnancy and gestational age at birth are psychiatric–neurological disorders, diabetes, asthma, sleep-related breathing disorders, and anemia, arterial hypertension [17,18]. In the pandemic context (SARS-CoV-2), pregnant women have a greater sensitivity to this infection, considering the physiological changes of the immune system at this stage. The main consequences of this disease associated with prematurity are represented by preeclampsia, gestational diabetes, and low birth weight [19].

Premature babies may experience both short-term and long-term complications. These can include challenges with breathing and feeding, as well as the risk of cerebral palsy and impairment in brain development later in life. Additionally, visual and hearing impairments can impact their overall prognosis. Preterm babies have important economic effects on individuals, families, and society. The survival rate of preterm babies in developed countries is higher compared to developing countries. The disparity may result from the superior neonatal care infrastructure and lower levels of psychosocial inequality found in higher-income nations compared to developing countries [14].

Considering the information above, our study analyzes more closely the premature births that took place in the period 2018–2022 in the “St. Apostol Andrei” County Emergency Clinic Hospital and aims to explore as widely as possible all available data on morbidity and mortality. We also want to evaluate potential maternal conditions to obtain a comprehensive understanding of the main causes and risk factors for preterm birth deaths. This study can be the basis for further action to prevent premature deaths.

## 2. Materials and Methods

The study was retrospective over 5 years (2018–2022). It was based on preterm infants (<37 weeks of gestation) born in the Constanta County Emergency Clinic Hospital, Romania. The cases were selected from the hospital database. All premature babies who died in the first year of life and whose necropsy was performed at the Constanța Pathological Anatomy Clinical Service were included in the study.

The causes of the deaths were determined after the autopsy. Both live-born premature and stillborns were externally examined by taking the characteristic measurements (birth weight in grams and length, cranial circumference, chest circumference, and abdominal circumference in centimeters). The internal examination involved examining each organ and taking samples for the histopathological examination from the meninges, brain, lungs, heart, stomach, mesentery, intestines, liver, spleen, kidneys, and adrenals.

According to the gestational age, 4 categories were defined: extremely premature infants (EPT) < 28 weeks, very premature infants (VPT) 28–31 weeks and 6 days, medium preterm infants (MPT) 32–33 weeks and 6 days, and late preterm infants (LPT) 34–36 weeks and 6 days [20]. According to birth weight, 3 categories were defined: extremely low birth weight (ELBW) <1000 g, very low birth weight (VLBW) 1000–1499 g, and low birth weight (LBW) 1500–2499 g. Normal birth weight (NBW) represents the range of 2500–4000 g and corresponds to a fourth category [21,22]. According to current legislation and World Health Organization/International Classification of Diseases (WHO/ICD) registration rules, stillbirths are reported as fetuses with a weight of at least 500 g, a gestational age of at least 22 weeks or a body length of at least 25 cm [3]. Survival was calculated in hours from birth to death. The APGAR score recorded immediately after birth (1 min score) was taken into account, having values from 0 to 10. Premature conception products that presented single or multiple malformations were also studied. Along with the previously mentioned fetal parameters, maternal data regarding age, number of pregnancies, parity, ailments at the time of presentation to the doctor, and the diagnosis at admission were also taken into consideration.

Statistical data analysis was performed in SPSS Statistics Version 26 (IBM Corporation, Armonk, NY, USA). Indicators of central tendency and variability were used. An analysis of univariate data was performed via Fisher’s exact test for categorical data, the Mann–Whitney U test, and the Kruskal–Wallis H test for continuous variables. To measure the association of the data, we used the Pearson correlation coefficient, and for the prediction of the response between variables, the Pearson regression. Receiver operating characteristic (ROC) and area under the curve (AUC) were used to establish the accuracy of the parameters. The sensitivity and specificity of the parameters are the optimal cut-off point as the value that maximizes the area under the ROC curve. Hazard ratios (HRs) were appreciated by using Cox regression analysis. Results were considered statistically significant at a *p*-value of <0.05.

All patients signed the informed consent at the time of hospitalization, and ethics approval was obtained from the local ethics commission (Ethics Commission of the Constanta County Emergency Hospital).

## 3. Results

**Figure 1:**
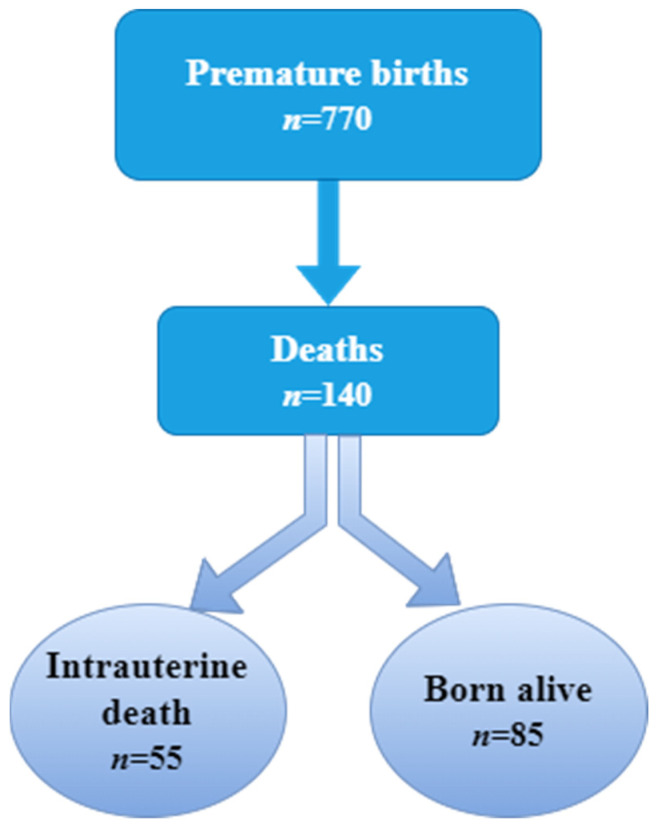
Flow chart with the studied batch.

In the period 2018–2022, 17,609 term births and 770 premature births were registered. Out of the total number, 303 deaths occurred, of which 140 were premature. Out of the 140 cases, 85 premature babies were born alive and died in less than a year of life. The other 55 died intrauterine and were born either naturally or by cesarean section (Figure 1).

**Figure 2:**
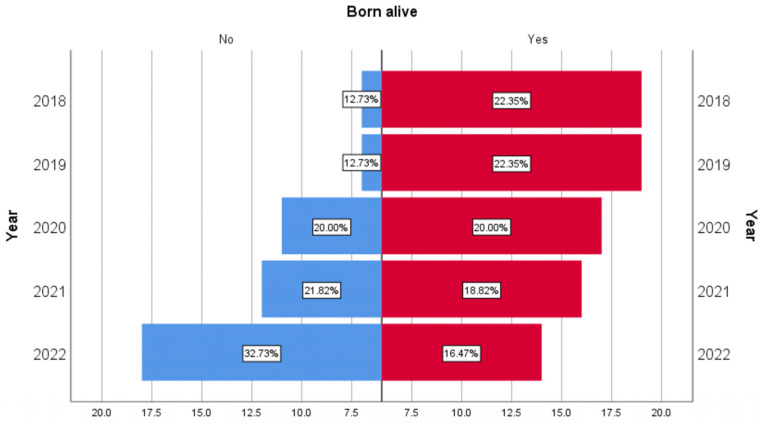
Graph showing the upward trend of intrauterine deaths of premature babies and the downward trend of deaths in the first year of premature babies born alive.

If, in the case of premature babies born alive, we observed a downward trend in the five years from 22.35% to 16.47%, in the case of stillborns, their evolution was on an upward slope from 12.73% to 32.73% (Figure 2).

Also, we observed a continuously increasing annual mortality rate, this being more evident in the case of stillborn premature babies. In the case of premature babies who died in the first year after birth, the mortality rate showed variations (Table 1).

A difference was observed between the degree of prematurity in relation to gestational age and viability at birth, so stillborn infants had an increased gestational age (MPT and LPT), while those born alive had smaller gestational age (EPT and VPT) (*p* < 0.001). Also, the same difference emerged between the degree of prematurity in relation to the weight and viability of the babies (*p* < 0.001). Therefore, the anthropometric parameters (cranial circumference and thoracic circumference) showed a higher value, in correspondence with the degree of maturation and fetal viability (*p* < 0.001 and *p* = 0.001, respectively) (Table 2).

In the case of premature babies born alive, the average survival time was 301.76 h, ±80.64 (1–4380 h). The most common APGAR score at 1 min was 1 (1–8). Increased gestational age was associated with an increased APGAR score (*p* < 0.001). Gestational age correlated directly proportionally with weight, length, cranial circumference, thoracic circumference, and abdominal circumference, suggesting harmonious development (*p* < 0.001). In addition, increased weight and abdominal circumference at birth were associated with longer infant survival (*p* = 0.023 and *p* = 0.002, respectively).

In the case of the degree of prematurity related to weight, a difference in survival was observed. This was decreased for VLBW followed by ELBW and increased for NBW followed by LBW (*p* = 0.046). In the case of the degree of prematurity related to the gestational age, no statistically significant differences were observed regarding survival (*p* = 0.981). Both in the case of the degree of prematurity related to weight, as well as that related to gestational age, statistically significant differences were observed regarding the APGAR score. A better score was found in LBW followed by NBW, LPT followed by VPT, and a lower score in ELBW followed by VLBW, EPT followed by MPT (*p* = 0.035 and *p* = 0.011, respectively) (Table 3).

The univariate analysis did not identify risk factors among the demographic aspects (sex, gestational age, APGAR score, degree of prematurity according to weight and term). Instead, the multivariate analysis of the data noted that ELBW (HR = 5.141, *p* = 0.009) and VLBW (HR = 4.177, *p* = 0.018) are risk factors involved in mortality.

Univariate analysis of anthropometric data identified protective factors: length (HR = 0.957, *p* = 0.027), chest circumference (HR = 0.937, *p* = 0.008), and abdominal circumference (HR = 0.922, *p* = 0.003). Multivariate analysis of anthropometric aspects identified cranial circumference as a mortality risk factor. For each centimeter, the hazard ratio was 1.134, with a *p*-value of 0.018. In contrast, the abdominal circumference remains a protective factor (for each cm HR = 0.901, *p* = 0.041).

In the case of premature stillborns, we identified the fact that gestational age was associated with weight, length, skull circumference, chest circumference, and abdominal circumference, suggesting a harmonious development of the products of conception (*p* < 0.001).

In 60.71% of the cases, maternal conditions were detected. The most common are pregnancy-induced hypertension (15.71%), cicatricial uterus (7.14%), and cervical insufficiency (5%). We observed statistically significant differences between the pathology of the mother and the viability of premature babies (*p* = 0.006). Thus, the presence of gestational diabetes and cervical insufficiency correlated with premature babies born alive, while the other pathologies were associated with premature babies born dead. The most common infectious causes were urinary tract infections (3 cases), HIV infection (2 cases), SARS-CoV-2 (1 case), syphilis (1 case), and acute cervicitis (1 case). In the case of the patient with SARS-CoV-2 infection, the fetus was stillborn (30 weeks, LBW) and presented pulmonary atelectasis and intestinal, hepatic, renal, and splenic hemorrhages.

At the time of hospitalization, some patients (10.71%) were provided with two diagnoses. The main diagnosis was established at the time of admission, and the secondary one was added after the birth of the fetus. The most common hospitalization main diagnoses were amniotic membrane rupture (15.71%), fetal distress syndrome (15%), and painful uterine contractions (14.29%). Other less frequent diagnoses added after birth were Rh incompatibility, nuchal cord, oligoamnios, polyhydramnios, placenta praevia, birth asphyxia, and malformation syndromes (11.43%). We observed a statistically significant difference between hospitalization diagnoses and viability of premature infants (*p* < 0.001). Thus, the diagnoses of painful uterine contractions, respiratory distress of the newborn, and ruptured amniotic membranes were found in live premature babies, while the absence of fetal movements, placental abruption, metrorrhagia, and intact membranes was associated with stillborn premature babies (Table 4).

In the case of premature babies born alive, we noticed that the increased gestational age was associated with the increased parity of the mothers (*p* = 0.022). Also, the increased parity of mothers correlated with increased anthropometric parameters (weight—*p* = 0.006, length—*p* = 0.001, cranial perimeter—*p* = 0.001, thoracic perimeter—*p* = 0.040, and abdominal circumference—*p* = 0.043).

The advanced age of the mother at the time of birth was associated with an increased number of pregnancies and an increased number of children born (*p* = 0.003 and *p* < 0.001, respectively). Increased parity was associated with premature births with low and very low birth weight (*p* = 0.019). Also, advanced age was associated with the presence of maternal conditions, especially gestational diabetes, pregnancy-induced hypertension, and cervico-isthmic incompetence (*p* = 0.032). We observed that a mother’s age of over 30 years is predictable for the development of pregnancy-induced hypertension (sensitivity 85.7% and specificity 65.4%, AUC = 0.784, *p* = 0.013).

Univariate and multivariate analyses did not identify risk factors regarding maternal data (mother’s age, personal pathological history, number of pregnancies, and parity).

In the case of premature stillborns, the advanced age of the mother was associated with the number of pregnancies (*p* < 0.001) and the number of births (*p* = 0.002), but it was not correlated with the presence of underlying pathology (*p* = 0.428).

Thanatogenesis in the case of premature babies born alive is summarized in Table 5.

**Figure 3:**
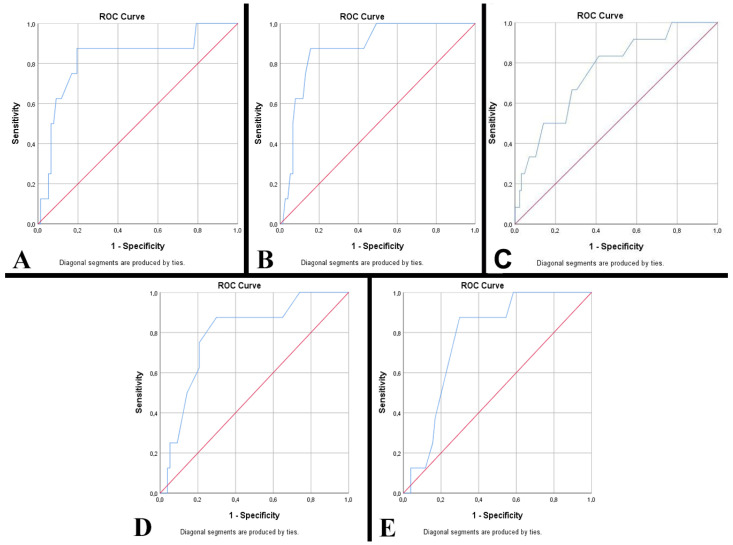
The ROC curve underlines the anthropometric changes in malformed premature babies (Red line represents the ROC curve for a random guess. The blue lines represent the distribution of the cases.). (**A**) Weight. (**B**) Length. (**C**) Cranial perimeter. (**D**) Chest circumference. (**E**) Abdominal circumference.

Malformed fetuses were identified in 7.27% of stillborn conception products. In the case of premature babies born alive, 9.41% were malformed. The presence of malformations was associated with increased parity (*p* = 0.005). An important aspect identified is represented by the predictability of anthropometric data on the development of malformed babies. A weight ≥ 1505 g at birth has increased predictability for malformations with a sensitivity of 87.50% and specificity of 80.50% (AUC = 0.825, *p* = 0.003) (Figure 3A). To these is added a length ≥ 41.50 cm—sensitivity of 87.50% and specificity of 84.40% (AUC = 0.876, *p* < 0.001), a cranial perimeter ≥ 28.50 cm—sensitivity of 87.50% and specificity of 77.90% (AUC = 0.840, *p* = 0.002), a chest circumference ≥ 25.75 cm—sensitivity of 87.50% and specificity of 77.10% (AUC = 0.793, *p* = 0.007), and an abdominal circumference ≥ 22.50 cm—sensitivity of 87.50% and specificity of 70.10% (AUC = 0.770, *p* = 0.012) (Figure 3B–E). Also, the presence of malformations was associated with a low (LBW) or normal birth weight (NBW) (*p* = 0.001), as well as with late (LPT) and medium (MPT) prematurity (*p* = 0.041).

Following the pathological examination of the necroptically examined organs, differences were identified between the diagnoses of some organs and the viability of premature babies. Thus, in the case of the pathology of the central nervous system, the correlation was observed between premature babies born alive and diagnoses of parenchymal hemorrhage, intraventricular hemorrhage, and other diagnoses (cerebral atrophy, periventricular leukomalacia, and brain abscesses), and between premature babies born dead and cerebral edema, autolytic changes, and the presence malformations (cerebellar hypoplasia, Dandy–Walker malformation, and total holoprosencephaly) (*p* = 0.035). In pulmonary pathology, the death of premature stillborns was associated with congestion and atelectasis (*p* < 0.001). In gastric pathology, we observed the association of premature babies born alive with upper digestive hemorrhage of unknown cause and those born dead with hemorrhagic gastritis (*p* = 0.001). In intestinal pathology, we found autolytic and hemorrhagic lesions prevalent in stillborn premature babies and the other diagnoses in the case of live births (*p* < 0.001). In spleen pathology, hemorrhagic lesions were associated with premature stillbirths (*p* = 0.007). In renal and adrenal pathologies, hemorrhages and autolytic lesions were associated with premature stillbirths (*p* = 0.022 and *p* = 0.007, respectively).

The detailed aspects of the diagnostics by organs can be viewed in the Table S1.

In the case of premature babies born alive, we noticed a difference in terms of aortic system diseases according to sex. Thus, in the case of females, we observed the presence of hypoplasia or aortic stenosis, while in the case of boys, the aortic system did not show any pathological changes (*p* = 0.041).

Some anthropometric aspects showed associations with death diagnoses. Thus, low cranial circumference correlated with the presence of cerebral intraventricular hemorrhage and cerebral parenchymal hemorrhage (*p* = 0.021), and low abdominal circumference correlated with cerebral atrophy and cerebral intraventricular hemorrhage (*p* = 0.018).

Regarding the APGAR score and the direct cause of death, significant statistical differences were observed. One child with a low score died from acute cardiorespiratory failure or multiple organ failure, while one with a high score died from sepsis or cerebral intraventricular hemorrhage (*p* = 0.006).

In pulmonary pathology, the presence of atelectasis was associated with increased mortality of premature babies, followed by hemorrhage and pneumonia (*p* = 0.009). Corroborating the personal pathological antecedents of the mother with fetal lung pathology, we identified associations between arterial hypertension and pulmonary hemorrhage, as well as between cervico-isthmic incompetence and neonatal bronchopneumonia (*p* = 0.024).

Some identified associations suggest a common mechanism of development of the lesional processes. These are represented by the association of brain parenchyma hemorrhage with adrenal hemorrhage (*p* = 0.047); the correlation of atrial and ventricular septal defects with the presence of hepatic hemorrhage, as well as the association between myocardial and hepatic dystrophy (*p* = 0.013); and association of cardiac and renal hemorrhage, as well as cardiac and renal dystrophy (*p* = 0.032). As expected, the presence of enterocolitis was associated with mesenteric adenopathies and fibrinous peritonitis (*p* = 0.001).

The univariate analysis performed on the pathology of each organ identified risk factors only in the case of pulmonary and renal pathology. Pulmonary atelectasis among pulmonary diseases represents a negative prognostic factor (HR = 54.391, *p* = 0.001) (Table 6). Polycystic kidney and renal edema represent negative prognostic factors and predictors of mortality (HR = 28.410, *p* = 0.004 and HR = 38.136, *p* = 0.002, respectively).

The multivariate analysis of pathology by region did not identify risk factors at the level of the cranial cavity (meninges and central nervous system) but identified risk factors at the level of the thoracic and abdominal cavity organs. The risk factors for the pathology of the thoracic organs (heart and lungs) observed in the multivariate analysis are pulmonary hemorrhage (HR = 12.874, *p* = 0.047), pulmonary atelectasis (HR = 66.583, *p* = 0.002), and cardiac dystrophy (HR = 3.190, *p* = 0.035) (Table 6). The risk factors for the pathology of the organs of the abdominal cavity (stomach, intestines, liver, spleen, kidneys, and adrenals) identified in the multivariate analysis are liver congestion (HR = 5.437, *p* = 0.036), liver infarction (HR = 34.002, *p* = 0.028), renal edema (HR = 28.605, *p* = 0.007), and polycystic kidney (HR = 52.381, *p* = 0.003).

In the case of premature stillborns, we observed an association between increased gestational age and autolytic and hemorrhagic heart lesions (*p* = 0.017). An interesting aspect derives from the fact that fetal lung damage presents statistically significant differences depending on the mother’s age. Thus, at the young age of the mother, pneumonia was identified, followed by atelectasis and pulmonary congestion (*p* = 0.048). The presence of maternal hypertension was associated with hemorrhagic gastritis of the fetus (*p* = 0.028), as well as with hemorrhagic lesions in the intestines (*p* = 0.049). Hemorrhagic lesions present at gastric level were associated with intestinal (*p* < 0.001) and splenic (*p* < 0.001) lesions, and liver lesions (dystrophy, congestion, and hemorrhage) were associated with the same type of lesions at the splenic and renal levels (*p* < 0.001 and *p* < 0.001, respectively).

## 4. Discussion

In Europe during 2020 were delivered 4.07 million alive babies, with a preterm birth rate of 8.7% (varying between 6.3 and 13.3%), while the rate of stillbirths—dead babies with a gestational age of at least 28 weeks—remained below 2.5 per 1000 total births [23,24,25]. Globally, 15% of premature babies are born before 32 weeks, 10.4% between 28 and 32 weeks, and 4.2% before 28 weeks of pregnancy [26]. In the study conducted by Kwasawneh W et al., the prematurity rate was 15.7%, with the majority of deaths occurring in babies born before the gestational age of 28 weeks [27]. In this study, most premature babies were born between 28 and 32 weeks of pregnancy (55.71%), followed by the late preterm ones (15.71%), extremely preterm (15%), and moderate preterm ones (13.57%).

Our data are in accordance with the data from the literature about the downward trend of cases of premature babies born alive, but also for the upward trend of stillborn babies. However, there is a particular aspect worth mentioning: our study covers the COVID-19 pandemic, even though it is not the primary focus. In studies conducted during the pandemic, there has been a decrease in the rates of premature fetuses stillborn or dying soon after birth. This observation suggests a potential role in reducing the death rates of the epidemiological measures taken during the pandemic period [25,28,29,30].

In the case of our study, the COVID-19 impact was not noticed because the patients who tested positive were transferred to another hospital that served SARS-CoV-2 infections during that period. The only case presented in the study could not benefit from the transfer, presenting in early preterm labor. For this reason, the study cannot evaluate the impact of COVID-19 in the case of premature deaths. Instead, SARS-CoV-2 infection in pregnancy was correlated with preeclampsia, premature birth, and stillbirth [19]. According to the study by Yang Z et al., the adverse evolutionary events of fetuses from mothers with COVID-19 included preterm birth (21.3%), fetal distress (10.7%), low birth weight (5.3%), stillbirth (1.2%), neonatal death (1.2%), and neonatal asphyxia (1.2%) [31]. These changes can also be explained by the fetal–placental malperfusion identified microscopically in COVID-19-positive cases [19]. According to the study carried out by Alcover N et al., the presence of placental changes that can be the cause of placental insufficiency and fetal hypoxia was observed in the cases of stillborn fetuses. Microscopic changes consist of chronic histiocytic intervillosis and massive perivillous fibrin deposits. Added to these is an exaggerated or dysregulated maternal inflammatory response [32]. Furthermore, these patients can develop disseminated intravascular coagulation, which leads to placental abruption [33].

In the case of premature stillborns, the advanced age of the mother was associated with the number of pregnancies and the number of births but did not correlate with the presence of underlying pathology. We noticed that women who are over 30 years of age are more prone to developing pregnancy-induced hypertension. This maternal condition was observed in over 60% of the mothers we studied, making it the most common pathological finding.

In terms of premature stillbirths, an upward trend has been observed globally over the years, with 2.6 million reported by the World Health Organization in 2015 and 1.7 million reported by the Global Burden of Disease Study in 2016 [2]. This aspect is particularly emphasized in our study, as we observed a steady increase in values during each year of research.

In the present study, we observed that the time to death of the patients was influenced by their birth weight. Thus, time to death was longer in normal and low birth weight cases compared to very low and extremely low birth weight (*p* = 0.046). According to the study conducted by Vilanova CS et al., the death rates of extremely low birth weight fetuses were 92.5%, decreasing with increasing gestational age [34]. The same downward trend was also observed in the research led by Ballot DE et al., in which the authors highlighted that the survival of patients with ELBW was 34.9% and increased to 85.8% in cases with VLBW [35].

Cnattingius S et al. studied the combined effect of gestational age and APGAR score on the risk of perinatal mortality and concluded that APGAR scores at 1 and 5 min provide reliable information on neonatal risk of premature death [36]. Park JH et al. observed that Apgar score at 5 min may be a better predictor of mortality from pulmonary causes in the first 7 postnatal days [37].

Many variable factors associated with the mother may be related to the risk of preterm birth: socioeconomic and demographic characteristics, behaviors and habits, health status, biological and reproductive characteristics, and pregnancy complications [38].

Any maternal infection can cause premature birth that results in the death of the fetus. In case of ascending infections, the lungs of the fetus become the primary organs affected with evidence of pneumonitis lesions [39]. In this study, gynecological infection was not present in cases of fetuses diagnosed with pneumonia or bronchopneumonia. The birth of stillbirths before 28 weeks of pregnancy is strongly associated with intrauterine bacterial infections, while the late premature birth of a stillborn fetus is unlikely to be determined by such a factor [39]. After analyzing the study group, we noticed that none of the cases born before 28 weeks of pregnancy had any previous or concurrent infection.

HIV infection can increase the risk of stillborn babies, especially in cases with a high viral load, the greatest risk for pregnant women with severe disease who do not receive treatment. Other causes of stillbirths are group B streptococcus infection and syphilis, causing an average value of 57,000 dead fetuses globally in 2015 [40]. In our study, the most common infectious causes were urinary tract infections, HIV infection, SARS-CoV-2, syphilis, and acute cervicitis. We identified one case each of maternal infection with SARS-CoV-2, group B Streptococcus, and Treponema pallidum, the birth of stillborn fetuses occurring between 29 and 31 weeks of pregnancy.

According to the study by Schindler T et al., the main causes of death identified, regardless of the gestational age studied, were intraventricular hemorrhage, acute respiratory pathology, and sepsis. They identified gestational age as the most important factor that predicted the death of patients, regardless of the cause of the event [41]. Similarly, in the present study, we observed similar causes of death, the most frequent being hemorrhages with different locations and acute respiratory pathology.

In the research carried out by Honein MA et al., malformations occurred twice as often in premature babies, and in very premature babies, their frequency was more than five times higher compared to full-term births. They observed the correlation between moderate prematurity and the central nervous system and cardiovascular damage. In addition, the authors identified esophageal and small intestine atresia as the main pathologies of the digestive tract associated with death [42]. In this study, brain and cardiovascular malformations contributed to the death of infants through determined hypoxic consequences. Moreover, we observed a pattern of anthropometric data in malformed children: the anthropometric data that should raise suspicion for the presence of a malformation are low or normal birth weight, late and average prematurity (MPT), a cranial circumference ≥28.50 cm, a chest circumference ≥25.75 cm, and a circumference abdominal ≥22.50 cm.

The term “encephalopathy of prematurity” is used to describe pathologies affecting the central nervous system and encompasses the profound impact of prematurity on the developing brain [43]. When considering stillbirth, brain abnormalities can range from common conditions such as leukomalacia or periventricular gliosis to more severe conditions such as intraventricular hemorrhage, cerebral infarctions, pontosubicular necrosis, and even spinal cord or brainstem necrosis. These pathologies lead to death, especially through hypoxia and infections [44]. In our study, a correlation was observed between premature babies born alive and the diagnoses of parenchymal hemorrhage and intraventricular hemorrhage. For preterm stillborns, we identified the association with cerebral edema and the presence of malformations.

Abdominal circumference is one of the best predictors of fetal growth, being one of the factors for evaluating intrauterine growth restriction (IUGR) [45]. Thus, to make this diagnosis, the value of the parameter must be below the 10th percentile [46]. Moreover, one of the risk factors for IUGR is maternal hypertension, a comorbidity also found in our study [45]. During the Doppler ultrasound evaluation, changes are observed in the middle cerebral arteries, with a decrease in oxygen pressure [47]. Moreover, hypoxia plays a key role in triggering vasodilation, which is essential for enhancing blood supply at this level [48,49]. On the other hand, the factors that influence the occurrence of intraventricular hemorrhage in premature infants are hypoxia and vasodilatation [50,51]. This constellation of events can explain the correlation between low abdominal circumference and intraventricular hemorrhages identified in the present study.

The global overview on neonatal death causes pins prematurity-related complications (including surfactant deficiency) at the top of the list (35%), together with intrapartum events (including birth asphyxia) and sepsis [52]. Respiratory distress syndrome of prematurity is a fundamental cause of morbidity and death in preterm-born babies. This is due to the lack or deficiency of pulmonary surfactant, which is normally produced after the 30th to 32nd week of pregnancy. Infants born before or around this stage will gradually experience respiratory distress with a varied range of symptoms. High airway pressures may also be required, exposing the patient to additional respiratory complications such as pneumothorax, interstitial lung emphysema, or pneumomediastinum [53]. Following our research, we identified the following lung pathologies as the cause of death of children born alive: pneumonia, bronchopneumonia, and hemorrhage, without identifying pathologies associated with therapeutic complications. Another frequent cause of death is aspiration pneumonitis, which is also the most avoidable cause [54]. Our study relates closely with the available data on the topic: the death of premature stillborns was associated with congestion (closely linked with maternal issues such as gestational hypertension) and atelectasis. The causes in terms of frequency were hemorrhage and pneumonia. By corroborating the maternal pathological antecedents with the fetal lung issues, we were able to observe associations between arterial hypertension and pulmonary hemorrhage, as well as between cervico-isthmic incompetence and neonatal bronchopneumonia. Cervico-isthmic incompetence and cervical insufficiency are responsible for 8% of recurrent spontaneous abortions [55]. Thus, the occurrence of an infection at this level can lead to the occurrence of fetal lung infections, the severity of which depends on the virulence of the etiological agent [39].

The immaturity of the gastrointestinal tract represents one of the major problems of neonatal care, taking into account that it has an essential role in the survival of newborns [56]. The main pathology at this level is necrotizing enterocolitis. This is the most common disease with a major contribution to the death of patients [56]. The most common factors that contribute to its occurrence are prematurity and low birth weight [56,57]. In our study, we found autolytic and hemorrhagic intestinal lesions prevalent in stillborn premature babies, which confirms the exposed available data. Also, we found an association between premature babies born alive and upper digestive hemorrhage of unknown cause and between those born dead with hemorrhagic gastritis, suggesting that there could be some joint cause or pathway for the phenomena. Hemorrhagic lesions were also described at splenic, renal, and adrenal levels, mostly linked with stillbirths, and an eventual fetomaternal hemorrhage should be considered. Fetomaternal hemorrhage consists of fetal blood leakage through a damaged placental barrier, resulting in fetal blood loss and hemolytic maternal effects [58]. Although fetal red blood cells typically enter the maternal bloodstream during most pregnancies, only significant occurrences pose a risk. This contributes to approximately 4.1% of antepartum stillbirths and an even higher rate of stillbirths at term [58,59].

In recent decades, great emphasis has been placed on prophylactic and therapeutic interventions for fetuses born before 34 weeks of pregnancy, but also for those with low birth weight [60]. Among these measures are the administration of antenatal corticosteroids, the prevention of infections, maintaining thermoregulation, assisting respiratory function, monitoring hemodynamic function, and taking care of neurological functions [26].

It should be noted that this research has certain limitations due to its retrospective nature and the difficulty of identifying a single cause of death for premature infants. Despite the extensive data panel that was evaluated, most situations were complex and involved multiple causes that could have contributed to the demise of the fetuses. The strengths lie in the extensive study of the data, with the creation of associations between them, starting from the medical characteristics of the mothers, the anthropometric data, up to the thanatogenesis and macroscopic necroptic diagnoses, which includes the malformations of all organs and systems. Also, this study complements the few data present in the literature, taking into account the small number of studies that holistically evaluate this information.

## 5. Conclusions

To conclude, the data collected and analyzed in our study support the existing literature. It was confirmed once more that the presence of atelectasis was associated with increased mortality of premature babies, and we found that pulmonary atelectasis among pulmonary diseases represents a negative prognostic factor. Also, we identified some associations suggesting a common mechanism of development for some lesional processes, such as brain parenchyma hemorrhage with adrenal hemorrhage); atrial and ventricular septal defects with the presence of hepatic hemorrhage, as well as the association between myocardial and hepatic dystrophy; association of cardiac and renal hemorrhage, as well as cardiac and renal dystrophy, all these leading to future optimization of the resources usage to predict and avoid possible preterm neonatal issues. These aspects open future research perspectives, including the possible common mechanism of these associations and the correlation of the presence of malformations with anthropometric data.

Our study emphasizes the importance of preventing premature deaths of fetuses through early diagnoses. We identified common causes of death associated with maternal pathology, which is often linked to hypoxia. Based on the data examined, it appears that it is possible to prevent thanatogenetic processes. To prevent in-utero or postpartum deaths, measures consisting of public education regarding the modifiable factors responsible for premature deaths, such as demographic factors and maternal infections, are required. Also, all possible ways must be ensured for easier access to specialized clinics, as well as a higher frequency of medical check-ups, in order to provide adequate prenatal care and birth screening methods. Thus, prenatal consultations are an effective and safe method for prevention, early diagnosis, and early stratification of births at high risk, as well as for the treatment of possible complications. It is crucial to take action at the first sign of suspicion of possible causes of premature death.

## Figures and Tables

**Table 1:** The number of deaths and the mortality rate during the years of study.

		Born Alive				Total	
		Yes		No			
		Deaths (*n*)	Mortality Rate (%)	Deaths (*n*)	Mortality Rate (%)	Deaths (*n*)	Mortality Rate (%)
Years	2018	19	10.92	7	4.02	26	14.94
	2019	19	12.18	7	4.49	26	16.67
	2020	17	10.49	11	6.79	28	17.28
	2021	16	9.94	12	7.45	28	17.39
	2022	14	11.97	18	15.38	32	27.35

**Table 2:** Demographic and anthropometric data of premature infants.

	Premature Born Alive	Premature Stillborns	*p*-Value
	(*n* = 85)	(*n* = 55)	
Gestational age (weeks)			<0.001
Average ± SE (min–max)	27.45 ± 0.37 (22–35)	30.73 ± 0.49 (22–35)	
Degree of prematurity according to gestational age (*n*, %)			<0.001
Extremely premature infants	17, 20%	4, 7.27%	
Very premature infants	55, 64.71%	23, 41.82%	
Medium preterm infants	7, 8.24%	12, 21.82%	
Late preterm infants	6, 7.06%	16, 29.09%	
Sex (*n*, %)			0.080
Female	30, 35.29%	28, 50.91%	
Male	55, 64.71%	27, 49.09%	
Weight (grams)			<0.001
Average ± SE (min–max)	1224 ± 84.54 (300–3975)	1632.36 ± 88.63 (290–3880)	
Degree of prematurity according to weight (*n*, %)			<0.001
Extremely low birth weight	50, 58.82%	8, 14.55%	
Very low birth weight	13, 15.29%	17, 30.91%	
Low birth weight	15, 17.65%	23, 41.82%	
Normal birth weight	7, 8.24%	7, 12.73%	
Length (cm)			<0.001
Average ± SE (min–max)	36.79 ± 0.65 (28–54)	40.45 ± 0.84 (24–54)	
Cranial circumference (cm)			0.001
Average ± SE (min–max)	26.31 ± 0.52 (19–46)	28.3 ± 0.53 (16–39)	
Chest circumference (cm)			0.006
Average ± SE (min–max)	23.88 ± 0.49 (12–38)	28.86 ± 0.63 (14–39)	
Abdominal circumference (cm)			0.113
Average ± SE (min–max)	21.7 ± 0.47 (14–38)	22.33 ± 0.53 (11–31)	

**Table 3:** Survival and APGAR score related to the degree of prematurity of premature babies born alive.

	Survival (h *, Mean ± SE)	*p*-Value	APGAR Score (Median)	*p*-Value
ELBW (*n* = 50) **	230.98 ± 88.27	0.046	1 (1–7)	0.035
VLBW (*n* = 13)	123.38 ± 36.02		2 (1–6)	
LBW (*n* = 15)	539.07 ± 278.91		3 (1–8)	
NBW (*n* = 7)	630.14 ± 451.01		2 (1–8)	
EPT (*n* = 17) ***	595.53 ± 302.15	0.981	1 (1–6)	0.011
VPT (*n* = 55)	252.33 ±80.81		2 (1–8)	
MPT (*n* = 7)	14.29 ± 64.89		1 (1–4)	
LPT (*n* = 6)	109.83 ± 32.20		4 (2–8)	

* hours; ** ELBW—extremely low birth weight; VLBW—very low birth weight; LBW—low birth weight; NBW—normal birth weight; *** EPT—extremely premature infants; VPT—very premature infants; MPT—medium preterm infants; LPT—late preterm infants.

**Table 4:** Maternal data relevant to premature births.

	Premature Born Alive	Premature Stillborns	*p*-Value
	(*n* = 85)	(*n* = 55)	
Mother’s age (years)			0.831
Average ± SE (min–max)	28.05 ± 0.74 (16–45)	27.82 ± 0.93 (16–46)	
Gestations			0.920
Average ± SE (min–max)	3.20 ± 0.28 (1–12)	3.25 ± 0.37 (1–12)	
Parity			0.254
Average ± SE (min–max)	1.92 ± 0.15 (1–9)	2.11 ± 0.19 (1–7)	
Maternal conditions (*n*, %)			0.006
No	60, 70.59%	25, 45.45%	
Hypertension	7, 8.24%	15, 27.27%	
Diabetes	3, 3.53%	1, 1.82%	
Cicatricial uterus	4, 4.71%	6, 10.91%	
Cervical insufficiency	6, 7.06%	1, 1.82%	
Cervico-isthmic incompetence	2, 2.35%	2, 3.64%	
Infectious causes	3, 3.52%	5, 9.09%	
Diagnosis at admission (*n*, %)			<0.001
Fetal distress	21, 24.71%	0, 0%	
Rupture of the amniotic membrane	17, 20%	5, 9.09%	
Painful uterine contractions	15, 17.65%	5, 9.09%	
Absence of fetal movements	1, 1.18%	17, 30.91%	
Intact amniotic membrane	7, 8.24%	12, 21.82%	
Placental abruption	7, 8.24%	7, 12.73%	
Preeclampsia	3, 3.53%	2, 3.64%	
Metrorrhagia	2, 2.35%	3, 5.45%	
Other diagnoses	12, 14.10%	4, 7.27%	

**Table 5:** Thanatogenesis of premature babies born alive.

Thanatogenesis	Diagnostics	Frequency (*n*, %)
Direct causes of death (I A)	Acute cardiorespiratory failure	72, 87.80%
	Sepsis	4, 4.88%
	Hemorrhagic disease of the newborn	2, 2.44%
	Multiple organ failure	2, 2.44%
	Cerebral intraventricular hemorrhage	2, 2.44%
Intermediate morbid conditions (I B)	Cerebral hemorrhage	10, 27.03%
	Pulmonary hemorrhage	9, 24.32%
	Cerebral edema	4, 10.81%
	Cerebral intraventricular hemorrhage	3, 8.11%
	Hydrocephalus	2, 5.41%
	Other diagnoses	9, 24.32%
Initial morbid conditions (I C)	Plurivisceral hemorrhages	22, 25.88%
	Cerebral hemorrhage	16, 18.82%
	Pulmonary hemorrhage	10, 11.76%
	Bronchopneumonia	7, 8.24%
	Pneumonia	7, 8.24%
	Other diagnoses	23, 27.06%
Other initial morbid conditions (I D)	Prematurity	72, 90%
	Plurimalformative syndrome	2, 2.50%
	Disseminated coagulation syndrome	1, 1.25%
	Congenital cardiac malformation	1, 1.25%
	Other diagnoses	4, 5%
Other important morbid conditions (II)	Prematurity	6, 27.27%
	Plurivisceral hemorrhages	4, 18.18%
	Enterocolitis	2, 9.09%
	Pneumonia	2, 9.09%
	Adrenal hemorrhages	2, 9.09%
	Other diagnoses	6, 27.30%

**Table 6:** Univariate and multivariate Cox regression of risk factors in lung and heart pathology.

Variables	Univariate Analysis			Multivariate Analysis		
	HR	CI 95%	*p*-Value	HR	CI 95%	*p*-Value
Lungs						
Normal	1			1		
Pneumonia	4.418	0.539–36.245	0.166	5.966	0.473–75.162	0.167
Bronchopneumonia	2.762	0.327–23.365	0.351	5.579	0.404–77.049	0.199
Hemorrhage	7.791	0.957–63.435	0.055	12.874	1.029–161.009	0.047
Congestion	5.023	0.286–88.147	0.270	6.585	0.261–166.141	0.252
Atelectasis	54.391	5.531–534.821	0.001	66.583	4.457–994.680	0.002
Pulmonary immaturity	5.421	0.444–66.231	0.186	8.880	0.503–156.911	0.136
Acute respiratory distress syndrome	7.754	0.440–136.718	0.162	14.173	0.582–344.865	0.104
Heart						
Normal	1			1		
Atrial septal defect	0.209	0.028–1.575	0.129	0.160	0.013–1.954	0.151
Ventricular septal defect	1.607	0.219–11.819	0.641	1.346	0.182–9.961	0.771
Dystrophy	2.850	1.092–7.442	0.032	3.190	1.085–9.377	0.035
Hemorrhage	1.261	0.797–1.996	0.322	1.367	0.840–2.225	0.208

## Data Availability

Dataset available upon request from the authors.

## Supplementary Materials

The following supporting information can be downloaded at https://www.mdpi.com/article/10.3390/clinpract14050144/s1, Table S1: The histopathological aspect of the examined organs.
