# Supplementary material for: Is It Possible to Prevent the Thanatogenetic Processes in Premature Babies?
Source: Clin Pract. 2024 Sep 2;14(5):1801–17. doi: 10.3390/clinpract14050144 (PMC11417739; doi:10.3390/clinpract14050144)
Supplement: Supplementary file 1 [file clinpract-14-00144-s001.zip › clinpract-3038580-supplementary.pdf]

Table S1. The histopathological aspect of the examined organs.

Region	Organ and diagnosis	Premature born alive (*n*, %)	Premature stillborns (*n*, %)	p-value
Cranial cavity	The meninges			
	Normal	20, 23.53%	13, 23.64%	0.567
	Meningitis	1, 1.18%	0, 0%	
	Edema	3, 3.53%	0, 0%	
	Hyperemia and hemorrhage	60, 70.59%	41, 74.55%	
	Sagittal sinus thrombosis	1, 1.18%	0, 0%	
	Autolysis	0, 0%	1, 1.82%	
	Central nervous system			
	Normal	7, 8.24%	5, 9.09%	**0.035**
	Edema	17, 20%	13, 23.64%	
	Parenchymal hemorrhage	41, 48.24%	22, 40%	
	Cerebral intraventricular hemorrhage	10, 11,76%	3, 5.45%	
	Malformations	2, 2.35%	2, 3.64%	
	Autolysis	0, 0%	4, 7.27%	
	Other diagnoses	8, 9.41%	6, 10.91%	
Thoracic cavity	The lungs			
	Normal	1, 1.18%	2, 3.64%	**<0.001**
	Pneumonia	20, 23.53%	2, 3.64%	
	Bronchopneumonia	8, 9.41%	0, 0%	
	Hemorrhage	46, 54.12%	14, 25.45%	
	Atelectasis	6, 7.06%	35, 63.64%	
	Congestion	1, 1.18%	2, 3.64%	
	Other diagnoses	3, 3.52%	0, 0%	
	Heart			
	Normal	46, 54.12%	26, 47.27%	0.458
	Dystrophy	5, 5.88%	4, 7.27%	
	Hemorrhage	31, 36.47%	20, 36.36%	
	Autolysis	0, 0%	2, 3.64%	
	Atrial septal defect	2, 2.35%	3, 5.45%	
	Ventricular septal defect	1, 1.18%	0, 0%	
Abdominal cavity	Stomach			
	Normal	73, 85.88%	41, 74.55%	**0.001**
	Upper digestive hemorrhage	4, 4.71%	0, 0%	
	Hemorrhagic gastritis	8, 9.41%	7, 12.73%	
	Autolysis	0, 0%	7, 12.73%	
	Bowels			
	Normal	72, 84.71%	41, 74.55%	**<0.001**
	Enterocolitis	7, 8.24%	0, 0%	
	Upper digestive hemorrhage	3, 3.53%	0, 0%	
	Blood infiltrate/hemorrhages	1, 1.18%	8, 14.55%	

	Necrosis	1, 1.18%	0, 0%	
	Occlusion	1, 1.18%	0, 0%	
	Autolysis	0, 0%	6, 10.91%	
	Mesentery			
	Normal	71, 83.53%	48, 87.27%	**<0.001**
	Adenopathies	6, 7.06%	1, 1.82%	
	Fibrinous peritonitis	8, 9.41%	0, 0%	
	Autolysis	0, 0%	6, 10.91%	
	Liver			
	Normal	18, 21.18%	10, 18.18%	0.063
	Hemorrhage	35, 41.18%	28, 50.91%	
	Congestion	14, 16.47%	7, 12.73%	
	Dystrophy	13, 15.29%	5, 9.09%	
	Autolysis	0, 0%	5, 9.09%	
	Other diagnoses	5, 5.88%	0, 0%	
	Spleen			
	Normal	41, 48.24%	16, 29.09%	**0.007**
	Dystrophy	7, 8.24%	7, 12.73%	
	Congestion	15, 17.65%	4, 7.27%	
	Hemorrhage	22, 25.88%	23, 41.82%	
	Autolysis	0, 0%	5, 9.09%	
	Kidney			
	Normal	34, 40%	11, 20%	**0.022**
	Dystrophy	7, 8.24%	4, 7.27%	
	Congestion	8, 9.41%	4, 7.27%	
	Hemorrhage	32, 37.65%	31, 56.36%	
	Malformations	3, 3.52%	1, 1.82%	
	Edema	1, 1.18%	0, 0%	
	Autolysis	0, 0%	4, 7.27%	
	Adrenal			
	Normal	40, 47.06%	17, 30.91%	**0.007**
	Hypertrophic	1, 1.18%	0, 0%	
	Hemorrhage	42, 49.41%	29, 52.73%	
	Autolysis	2, 2.35%	9, 16.36%	
More	Aortic system			
	Normal	82, 96.47%	55, 100%	0.708
	Hypoplasia of the aorta	1, 1.18%	0, 0%	
	Aortic stenosis	2, 2.35%	0, 0%	
	Fluid effusions/collections (24.3%)			
	Hydrocephaly	13, 48.15%	5, 71.43%	0.629
	Pleurisy	5, 18.52%	0, 0%	
	Pericarditis and pleurisy	2, 7.41%	1, 14.29%	
	Pericarditis	3, 11.11%	0, 0%	
	Chylopericardium	1, 3.70%	0, 0%	

	Ascites<br>Anasarca	1, 3.70%<br>2, 7.41%	1, 14.29%<br>0, 0%	
